# Studies of Wilms’ Tumor (*WT1*) Gene Expression in Adult Acute Leukemias in Singapore

**Published:** 2007-08-08

**Authors:** Che Kang Lim, Yeow Tee Goh, William Y. K. Hwang, Liam Pock Ho, Li Sun

**Affiliations:** 1 Department of Clinical Research; 2 Department of Haematology; 3 Department of Pathology, Singapore General Hospital, Singapore 169608

**Keywords:** WT1, adulthood acute leukemia, gene expression, HLA-A

## Abstract

Biomarkers provide certain values for diagnosis, monitor treatment efficacy, or for the development of novel therapeutic approach for particular diseases. Thus, the identification of specific of biomarkers for specific medical problems, including malignant diseases may be valuable in medical practice. In the study, we have used the Wilms’ tumor gene (*WT1*) as a biomarker to evaluate its expression in local adult patients with newly diagnosed acute leukemia, including both acute myeloid and lymphoid leukemias (AML and ALL).

**Aim:**

To investigate *WT1* gene expression in adult patients with acute leukemia at diagnosis.

**Methods:**

Eighteen patients with acute leukemia diagnosed at Singapore General Hospital, Singapore, between September, 2004 and July, 2005 were included in this study. There were fifteen AML and three ALL cases aged from 18 to 71 years old. Total RNA and DNA was extracted from peripheral blood mononuclear cells (PBMCs). Expression of *WT1* was detected by nested reverse-transcription polymerase chain reaction (Nested RT-PCR). K562, and 3T3 cells were used as positive- and negative-controls. The results were revalidated using real-time PCR. HLA-A genotyping was performed using sequence specific oligonucleotide polymorphism (SSOP) analysis.

**Results:**

*WT1* gene was exclusively expressed in all eighteen, including three ALL and fifteen AML, patients. In contrast with *WT1* gene, the HLA-A genotyping was remarkably heterogeneous in these patients.

**Conclusions:**

*WT1* gene expression was observed in local patients with acute leukemia at diagnosis. It may be used as a potential molecular marker for diagnosis, clinical progression of the diseases or monitoring the response to treatment, as well as a target for the development of novel therapeutic approaches.

## Introduction

Acute myeloid leukemia in adults is a common and lethal malignant disease. Despite the tremendous efforts in the improvement of treatment in recent years, the survival of acute leukemia in adults remained poor. The Wilms’ tumor (*WT1*) gene, located at chromosome 11p13 (1), was identified as a gene responsible for Wilms’ tumor, a kidney neoplasm of childhood (2). The *WT1* gene is encoded by 10 exons with different transcripts that subjects to alternative splicing. *WT1* gene encodes proteins isoforms with molecular masses ranging from 48 to 54 kDa with four zinc finger motifs. *WT1* gene plays multiple and important roles in cell biology, such as cell and tissue development, cell proliferation, differentiation, and apoptosis (3,4). It has been classified as a tumor suppressor gene—encoding a transcription factor. Expression of *WT1* has been observed in different types of solid cancers, such as ovarian cancer, mesothlioma of the lung, melanoma, breast cancer, as well as in Wilms’ tumor (5,6). It has been reported that the Wilms’ Tumor Gene (*WT1*) is expressed in leukemia blasts, irrespective of the subtypes of acute leukemia (7,8, 9). Early report showed that *WT1* antisense oligonucleotides could induce apoptosis in myeloid cell lines (10). In recent years, it has been found that *WT1* could be used as a molecular marker to generate specific cytotoxic T cells (CTL) against leukemia cells (11). The anti-leukemia activity of *WT1*-induced CTLs are reported to be HLA-A restricted and has been used as adoptive immunotherapy in some small scale clinical trials in patients with acute leukemia (12).

The current study was designed to investigate the *WT1* gene expression status in local adult patients with acute leukemia. It was believed that this was the first such study ever reported from Singapore. We were also exploring the possible clinical application of *WT1* as a molecular marker in adult patients with acute leukemia.

## Materials and Methods

### Sample collection and cell process

Peripheral blood (PB) was collected from eighteen consecutive adult patients with acute leukemia at diagnosis with informed consents and the approval of hospital’s Ethics Committee. Diagnosis for each patient was made according to the FAB criteria (13). PB was also obtained from healthy volunteers and used as normal controls. Mononuclear cells (MCs) were isolated using Ficoll-Hypaque (Amersham Pharmacia Biotech, Upsala, Sweden) density gradient and centrifugation with 2,000 RPM at 20 °C for 20 minutes. PBMCs were collected from the interface of the density gradient separation for RNA and DNA extraction.

### DNA and RNA purification

Total RNA was isolated using the RNeasy kit (Qiagen, Valencia, CA, U.S.A.) according to manufacturer’s protocol. Genomic DNA was isolated from mononuclear cell using a QIAamp Blood Mini Kit according to the manufacturer’s instruction (Qiagen, Valencia, CA, U.S.A.). Total RNA was used for nested RT-PCR and real-time PCR while DNA was used for HLA typing assay.

### Nested RT-PCR (reverse-transcription PCR)

One step nested reverse transcription polymerase chain reaction (Nested RT-PCR) was performed by using a one-step RT-PCR kit. (Qiagen, Valencia, CA, USA) *WT1* exon 1–4 was amplified using forward (5′-CCTACCTGCCC AG CTGCCTC-3′) and reverse (5′-CTCCTAAGTTCATCTGATTCC-3′) primers for 20 cycles (annealing temperature 56 °C), followed by nested PCR (forward: 5′-AGAGCCAGCCCGCTATTCG-3′; and reverse: GGTCATGCATTCAAGCTGG-3′ primers) for 30 cycles (annealing temperature 58 °C). The expected size for the PCR amplification was 284 bp(11). Amplifications were performed with the GeneAmp PCR system 9700 (Applied Biosystem, Foster City, CA, U.S.A.).

### Real-time PCR

cDNA synthesis reaction was performed with 1 μg of total RNA in a total volume of 20 μL containing 200 units M-MLV (moloney murine leukemia virus) reverse transcriptase (Invitrogen, Carlsbad, CA, U.S.A.), 1x First-Strand Buffer (50 mM Tris-HCl (pH 8.3) 75 mM KCl, 3mM MgCl_2_), 1mM dNTPs, 10 mM DTT, 5 mM random primers and incubated at 42 °C for 1hr. The reaction was inactivated by heating 90 °C for 10 min Real-Time PCR(14) was performed in a MicroAmp optical 96-well plate with 4 μL of the cDNA solution, 0.5 μl of forward and reverse primers, 8μL of dH_2_O and 12.5 μL of 2X SYBR Green Mix (Qiagen, Valencia, CA, U.S.A.). The sequences of forward and reverse primers for the semi-quantitative measurement of *WT1* expression were as follows:

5′-GATCCTGGACTTCCTCTTGCT-3′ (forward);

5′-CTGCTCTGGCTGCTGTAGG-3′ (reverse).

The reaction mixture was heated at 50 °C for 2 minutes and then at 95 °C for 15 minutes to activate the polymerase. PCR was then performed using an ABI Prism 7700 Sequence Detector System (Applied Biosystem, Foster City, CA, U.S.A.) with 40 cycles, one of which consisted of denaturation at 94 °C for 15 seconds, and annealing at 56 °C for 30 seconds followed by extension at 72 °C for 30 seconds. The *WT1* mRNA was assessed using the relative standard curve method (15).

### HLA-typing analysis

20 to 30 ng of genomic DNA was amplified using the Dynal RELI^™^ SSO HLA-A typing kit (Dynal Biotech GmbH, Hamburg, Germany). PCR was performed strictly following the manufacturer instructions. In brief: PCR was performed in a total volume of 50 μL with the following steps: 15 seconds at 95 °C, 45 seconds at 60 °C, 15 seconds 72 °C and hold for 5 minutes at 72 °C. The PCR was performed using the GeneAmp PCR system 9700 (Applied Biosystem, Foster City, CA, U.S.A.). The probe hybridization and strip detection were also carried out following the manufacture’s instructions (Dynal RELI^™^ SSO HLA-A Typing kit, Dynal Biotech, Germany). The HLA-A typing for the patient samples was interpreted using the pattern-matching program provided by Dynal (Dynal Biotech GmbH, Hamburg, Germany). HLA-A typing was performed in 15/18 (83.3%) patients and the results were shown in Table 1.

## Results

### Nested RT-PCR

**Figure 1:**
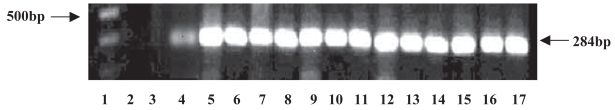
Nested PCR for WT1 expression.

A 284bp band for the exon 1–4 of *WT1* gene was detected in all 18 patients. The nested RT-PCR results from selected patients (Patients 1 to 12 shown in Table 1**)** are summarized in Figure 1. In comparison to normal PBMCs, *WT1* mRNA was detected at higher levels in all 18 patients with acute leukemia (AML), including 3 patients with acute lymphoblastic leukemia (ALL) and 15 patients with acute myeloid leukemia (AML). However, no densitometry was applied to compare the intensity of RT-PCR products in this study.

### Real-time PCR

**Figure 2:**
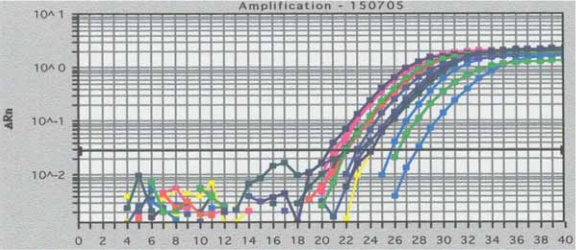
RT-PCR results from selected patients numbred in Table 1 which RT-PCR results shown in Figure 1. The Ct values are provided as follows: **Table:** 

Real-time PCR was performed as confirmational experiments to verify the *WT1* mRNA over expression in the patients with acute leukemia. The *WT1* mRNA levels from all 18 patients with acute leukemias was 41.39 ± 36.20% in comparison to K562 cells. Real-Time PCR results from 1 patient with ALL and 5 with AML are shown in Figure 2. The Ct values of the real-time PCR are indicated in the insert of Figure 2.

### HLA-A typing

Unlike the universal expression of *WT1* gene, the HLA genotyping in this cohort of adult acute leukemia patients was heterogeneous. The results of HLA-A typing of 15/18 patients from this study were summarized in Table 1. Coincidentally, all 3 patients with ALL showed identical HLA-A typing. The HLA-A typing appeared to be more heterogeneous in patients with AML.

Although there was no correlation observed between subtypes of AML and HLA-A typing, it was noticeable that 7/12 (58.3%) AML patients (patients number 4, 5, 6, 7, 8, 9 and 11) showed HLA-A2 genotype.

## Discussion

*WT1* gene has been shown to be universally expressed in various types of malignant blood disorders, such as acute leukemia, chronic granlocytic leukemia, multiple myeloma, etc (16,17,18,19). A number of reports have shown that *WT1* gene could be used as a molecular marker for diagnosis, monitoring clinical progress in acute leukemia, and more importantly, as a molecular target for adoptive immunotherapy (8,9,10).

The results from current study have demonstrated that the *WT1* gene was “universally” expressed at diagnosis in a small cohort of local adult patients (18/18) with acute leukemia, including both AML and ALL, regardless of lineages of the leukemia cell origin. The results are in consistent with that reported in the literature (16, 17,18,19). However, follow-up study could not be performed, largely due to the small numbers of circulating residual leukemia blasts post-induction chemotherapy.

By using a more sensitive method, i.e. real-time PCR, we found that the *WT1* mRNA was expressed at a lower level in the normal PBMCs (Figure 2). In contrast, the expression level in the leukemia cells was much higher than that in normal blood cells. This observation was similar to that reported previously (20,21,22). Although the sequence of the *WT1* mRNA was not examined in the current study, it is likely that the *WT1* gene related to leukemia to be a wild type rather than mutation (23).

The current study has shown that HLA-A typing was heterogeneous in adult acute leukemia patients. Although all three ALL patients showed identical HLA-A typing (Table 1), this was likely to be a coincidental observation and it could not draw any conclusion between a particular HLA allele and leukemogenesis in adulthood acute lymphoblastic leukemia. In comparison to ALL, the HLA-A typing in AML patients was more heterogenous. HLA-A2 was seen in 7/12 (58.3%) of the AML samples that had been HLA-A typed. However, no correlation between HLA-A typing and *WT1* mRNA levels in AML was observed. The results of HLA-A typing would be helpful for further evaluating the anti-leukemia activity in the cytotoxic T-lymphocyte (CTL) to be generated using leukemia cells from the patients. Studies in recent years by a number of reports have demonstrated that the *WT1* protein-induced specific anti-leukemia activity by cytotoxic T lymphocytes (CTL) was HLA-A2-restricted. Colony formation by normal bone marrow cells of HLA-A2-positive patients were not inhibited by the *WT1* leukemia-specific CTL (10, 24, 25). All these reports have demonstrated that *WT1* proteins or peptides could be used as molecular targets for the development of specific cell based anti-leukemia therapy. *WT1* peptides as vaccine for anti-leukemia immunotherapy will be evaluated in our local patients in the future.

A recent report by Hossain et al has described the assessment of different *WT1* transcripts, especially a 2.3 Kb short *WT1* transcript (s*WT1*) which encodes a protein of ~35–37 kDa, was over-expressed in leukemia (26). Further study should be embarked to elucidate the *WT1* isoforms in our patient population.

In conclusion, *WT1* gene was universally expressed in local adult patients with acute leukemia at diagnosis in Singapore. It may be used as a potential molecular marker for diagnosis, monitoring the clinical progress, the response to treatment, as well as a target for the development of novel therapeutic approaches.

## Figures and Tables

No.	Samples	Ct Value	No.	ID	Ct Value
1	Blank	40	9	Patient 6 (AML/M2)	22.13
3	Negative control (3T3)	39.82	11	Patient 7 (AML/GCL → AML)	24.1
2	Positive control (K562)	22.93	10	Patient 8 (AML/M5b)	22.03
4	Normal control	27.92	12	Patient 9 (AML/M5a)	25.67
5	Patient 1 (ALL)	21.1	13	Patient 10 (AML/M5)	21.44
6	Patient 2 (ALL)	23.01	14	Patient 11 (AML/M1)	23.52
7	Patient 4 (AML/M2)	21.95	15	Patient 12 (AML/M2)	23.22
8	Patient 5 (AML/M1)	24.14	16	Patient 13 (AML/M2)	20.62

**Table 1:** *WT1* Expression Profile and HLA-A Typing in Patients with Acute Leukaemia.

Patients	Acute Leukaemia (Sub-types)	WT1 Expression	HLA-A Typing
1	ALL	Yes	*A-03201
2	ALL	Yes	*A-03201
3	ALL	Yes	*A-03010
4	AML (M2)	Yes	*A-020101
5	AML (M1)	Yes	*A-020501
6	AML (M2)	Yes	*A-020101
7	AML (CGL → AML)	Yes	*A-020501
8	AML (M5b)	Yes	*A-020101
9	AML (M5a)	Yes	*A-020501
10	AML (M5)	Yes	*A-240201
11	AML (M1)	Yes	*A-020101
12	AML (M2)	Yes	*A-240201
13	AML (M2)	Yes	*A-030101
14	AML (M7)	Yes	*A-030101
15	AML (M1)	Yes	*A-030101
16	AML (M3)	Yes	NT
17	AML (M4)	Yes	NT
18	AML (3v)	Yes	NT

**ALL:** Acute lymphoblastic leukaemia; **AML:** Acute myeloid leukaemia; **CGL:** Chronic granulocytic leukaemia; **HLA:** Human leukocyte antigen, only HLA haplotyping could be confirmed in the patients. **NT:** Not tested.
